# Adjustable Thermal Expansion Properties in Zr_2_MoP_2_O_12_/ZrO_2_ Ceramic Composites

**DOI:** 10.3389/fchem.2018.00347

**Published:** 2018-08-14

**Authors:** Hongfei Liu, Weikang Sun, Xiang Xie, Lu Yang, Zhiping Zhang, Min Zhou, Xianghua Zeng, Xiaobing Chen

**Affiliations:** ^1^Department of Microelectronics, School of Physical Science and Technology, Yangzhou University, Yangzhou, China; ^2^Department of Electrical and Mechanical Engineering, Guangling College, Yangzhou University, Yangzhou, China

**Keywords:** ceramics, Zr_2_MoP_2_O_12_, ZrO_2_, composites, thermal expansion control

## Abstract

Zr_2_MoP_2_O_12_/ZrO_2_ composites were successfully synthesized by the solid state method in attempt to fabricate the near-zero thermal expansion ceramics. The phase composition, micromorphology and thermal expansion behavior of the Zr_2_MoP_2_O_12_/ZrO_2_ composites with different mass ratios were investigated using X-ray diffraction, scanning electron microscopy and thermal mechanical analysis. Results indicate that Zr_2_MoP_2_O_12_/ZrO_2_ composites can be prepared by pre-sintering at 500°C for 3 h and then sintering at 1050°C for 6 h. The resulting Zr_2_MoP_2_O_12_/ZrO_2_ composites consisted of orthorhombic Zr_2_MoP_2_O_12_ and monoclinic ZrO_2_. With increasing content of Zr_2_MoP_2_O_12_, the Zr_2_MoP_2_O_12_/ZrO_2_ ceramics became more compact and the coefficient of thermal expansion decreased gradually. Zr_2_MoP_2_O_12_/ZrO_2_ composites show an adjustable coefficient of thermal expansion (CTE) from 5.57 × 10^−6^ K^−1^ to −5.73 × 10^−6^ K^−1^ by changing the mass ratio of Zr_2_MoP_2_O_12_ and ZrO_2_. The Zr_2_MoP_2_O_12_/ZrO_2_ composite with a mass ratio of 2:1 showed near zero thermal expansion, and the average linear thermal expansion coefficient is measured to be 0.0065 × 10^−6^ K^−1^ in the temperature range from 25 to 700°C.

## Introduction

It is well known that the vast majority of materials expand on heating. However, some materials shrink as the temperature rises and display negative thermal expansion (NTE). NTE materials have attracted considerable attention due to the anomalous phenomenon and their potential application in controlled thermal expansion composites and other areas (Chen et al., 2015). Control of thermal expansion is crucial to many applications. Mismatch of thermal expansion in component materials of high-precision device may result in serious problems, such as mechanical destruction and positional deviation. An easy method to prepare the material with near-zero or low thermal expansion is combining NTE materials with positive thermal expansion materials (Liu et al.^2012^; 2012a; Gao et al., 2016; Zhang et al., 2017). Materials displaying zero or low thermal expansion are both dimensionally stable and highly resistant to thermal shock.

The longtime leading NTE exemplar was ZrW_2_O_8_. Cubic ZrW_2_O_8_ exhibits strong isotropic NTE over a wide temperature range (Mary et al., 1996; Nishiiyama et al., 2006; Kanamori et al., 2008; Banek et al., 2010; Liu et al., 2011, 2012b). With the goal of thermal expansion control, several studies of the composites containing ZrW_2_O_8_ have been reported, such as ZrW_2_O_8_/ZrO_2_ (Lommens et al., 2005; Yang et al., 2007), ZrW_2_O_8_/Cu (Yilmaz, 2002), ZrW_2_O_8_/Al (Wu et al., 2013), ZrW_2_O_8_/cement (Kofteros et al., 2001), ZrW_2_O_8_/PI, and ZrW_2_O_8_/epoxy (Sullivan and Lukehart, 2005; Yang et al., 2010). The thermal expansion coefficient of composites can be effectively controlled by using ZrW_2_O_8_ as NTE filler, however, the cubic ZrW_2_O_8_ is metastable at room temperature and needs to be quenched after sintering at 1200°C. Meanwhile, ZrW_2_O_8_ undergoes a structural phase transition from α -ZrW_2_O_8_ to *β* -ZrW_2_O_8_ at around 160°C and the coefficient of thermal expansion will decrease from about −8.8 × 10^−6^ K^−1^ to −4.9 × 10^−6^ K^−1^. In addition, when ZrW_2_O_8_ was heated to about 740°C in air, it will decompose into ZrO_2_ and WO_3_ (Mary et al., 1996; Nishiiyama et al., 2006; Kanamori et al., 2008; Banek et al., 2010; Liu et al., 2011, 2012b). Quenching, thermal decomposition and the abrupt change of thermal expansion are disadvantageous for composite design.

Orthorhombic Zr_2_MoP_2_O_12_, a member of A_2_M_3_O_12_ family, has received widespread attention in recent years. It shows stable NTE and its average linear expansion coefficient is −4.5 × 10^−6^ K^−1^ over a broad temperature range from −264 to 1050°C. What's more, it overcomes all the limitations of ZrW_2_O_8_ discussed above, suggesting its potential use for fabricating near-zero or low thermal expansion materials (Cetinkol et al., 2009; Isobe et al., 2016). ZrO_2_ ceramics has been widely used in optical, electrical and high temperature fields. The average linear thermal expansion coefficient of ZrO_2_ is about 10 × 10^−6^ K^−1^(Lommens et al., 2005; Yang et al., 2007). The absolute values of thermal expansion coefficient of ZrO_2_ and Zr_2_MoP_2_O_12_ are thus similar but have opposite signs, suggesting that these materials are good candidates for the preparation of ceramic composites with tunable CTEs. In this work, a new series of Zr_2_MoP_2_O_12_/ZrO_2_ composites were synthesized by solid state method with the goal of tailoring the thermal expansion. The effects of mass ratio between Zr_2_MoP_2_O_12_ and ZrO_2_ on the phase composition, microstructure, and thermal expansion coefficient of the Zr_2_MoP_2_O_12_/ZrO_2_ ceramic composites were also investigated.

## Experimental

### Sample preparation

Zr_2_MoP_2_O_12_, ZrO_2_, and Zr_2_MoP_2_O_12_/ZrO_2_ composites (mass ratios: 1:2, 1:1, 2:1) were prepared using stoichiometric amounts of ZrO_2_ (purity ≥99.95%, metals basis), MoO_3_ (purity ≥99.99%, metals basis), and NH_4_H_2_PO_4_ (purity ≥99.5%, metals basis). A summary of samples prepared can be found in Table 1. The starting compounds were mixed in ethanol using ball milling for 6 h to form a uniform mixture and dried at 80°C, then the mixtures were pre-heated at 500°C for 3 h. The mixtures, under 50 MPa, were cold pressed into pellets which were 7 mm in diameter and about 2 mm in thickness. Finally, the pellets were calcined at 1050°C in air for 6 h and cooled down in the furnace. In the solid-state synthesis, the following reactions may take place:

(1) $$\begin{array}{l} {\text{ZrO}}_{2}+{\text{MoO}}_{3}+{\text{2NH}}_{4}{\text{H}}_{2}{\text{PO}}_{4}\to {\text{ZrP}}_{2}{\text{O}}_{7}+{\text{2NH}}_{3}↑\\ \;+{\text{3H}}_{2}\text{O}+{\text{MoO}}_{3}(500^{{}^{\circ}}\text{C}) \end{array}$$

(2) $${\text{ZrO}}_{2}+{\text{MoO}}_{3}+{\text{ZrP}}_{2}{\text{O}}_{7}\to {\text{Zr}}_{2}{\text{MoP}}_{2}{\text{O}}_{12}(1050^{{}^{\circ}}\text{C})$$

(3) $$\begin{array}{l} \text{Or}:(1+\text{x}){\text{ZrO}}_{2}+{\text{MoO}}_{3}+{\text{ZrP}}_{2}{\text{O}}_{7}\to {\text{Zr}}_{2}{\text{MoP}}_{2}O_{12}\\ \;+{\text{xZrO}}_{2}(1050^{{}^{\circ}}\text{C}) \end{array}$$

**Table 1:** Synthesis conditions for ZrO_2_, Zr_2_MoP_2_O_12_, and Zr_2_MoP_2_O_12_/ZrO_2_ ceramics.

**Mass ratios of Zr_2_MoP_2_O_12_:ZrO_2_**	**m(ZrO_2_)/g**	**m(MoO_3_)/g**	**m(NH_4_H_2_PO_4_)/g**
0:1	10	0	0
1:2	9.8523	1.0824	1.7290
1:1	8.7776	1.6227	2.5930
2:1	7.7038	2.1635	3.4576
1:0	4.1655	2.4330	3.8895

In this work, the 2:1 Zr_2_MoP_2_O_12_/ZrO_2_ composite was also prepared using the pure Zr_2_MoP_2_O_12_ and ZrO_2_ as raw materials, and the synthetic process is the same as that of the above process.

### Experimental techniques

Identification of the different phases presented in the samples was performed using powder X-ray diffraction (PXRD) on a Shimadzu XRD 7000 with Cu Ka radiation. Data were collected at 40 kV and 35 mA, with a scanning speed of 5°/min over an angular range of 10–60°. The micromorphologies of the samples were observed using a scanning electron microscopy (SEM, TESCAN VEGA3). The elemental composition of the samples were analyzed using energy-dispersive X-ray spectrometry (EDX, Bruker XFlash 6160) as well.The CTEs of the samples were measured by thermal mechanical analyzer (TMA/SS, Seiko 6300) using a heating rate of 5°C/min from room temperature to 700°C in air.

## Results and discussion

### XRD analysis

**Figure 1:**
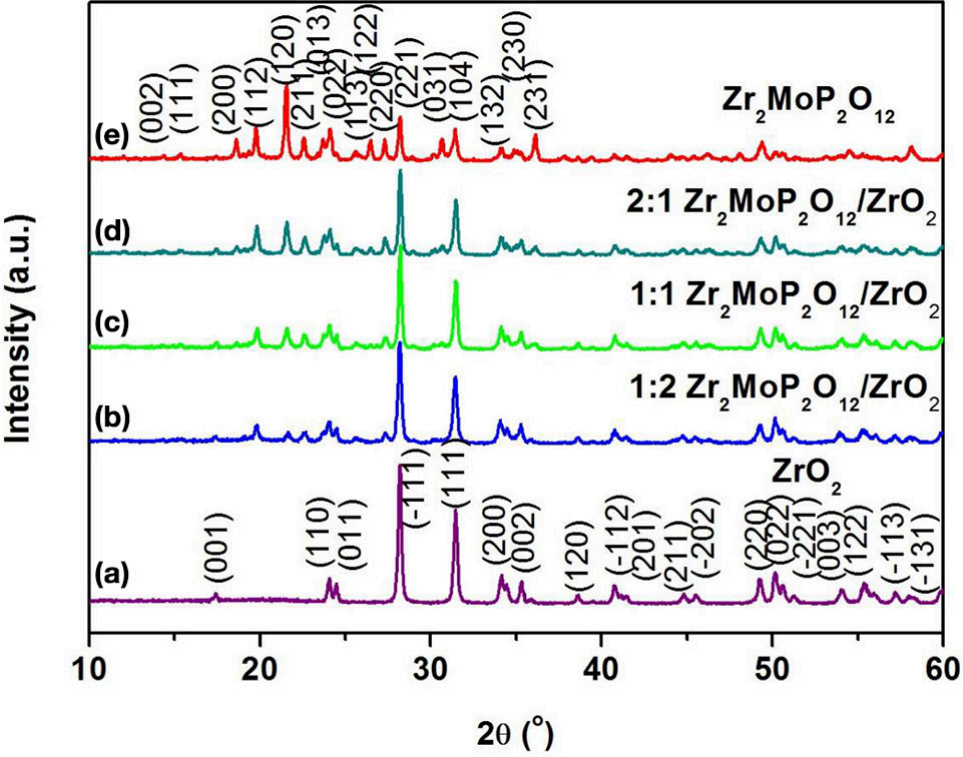
XRD patterns of the obtained ZrO_2_ ceramics, Zr_2_MoP_2_O_12_ ceramics and Zr_2_MoP_2_O_12_/ZrO_2_ composites with different mass ratios sintered at 1050°C for 6 h.

Figure 1 shows typical XRD patterns of the Zr_2_MoP_2_O_12_/ZrO_2_ (mass ratios 1:2, 1:1, and 2:1) composites in addition to those of the pure Zr_2_MoP_2_O_12_ and ZrO_2_ ceramics obtained under the same preparation condition. Figure 1a shows typical powder XRD pattern of pure ZrO_2_ ceramics sintered at 1050°C for 6 h, all the observed reflections could be well indexed and attributed to monoclinic ZrO_2_ in agreement with JCPDS card number 65–1,023. The typical powder XRD pattern of pure Zr_2_MoP_2_O_12_ specimen sintered at 1050°C for 6 h is shown in Figure 1e, all diffraction peaks match those expected for orthorhombic Zr_2_MoP_2_O_12_, which agrees with literature reports (Cetinkol et al., 2009; Isobe et al., 2016). No impurity phases were detected, confirming the purity of the two products. The PXRD patterns of the Zr_2_MoP_2_O_12_/ZrO_2_ (mass ratio: 1:2, 1:1, and 2:1) composites were shown in Figures 1b–d, it can be seen that all the diffraction peaks of the specimens could be indexed as both monoclinic ZrO_2_ and orthorhombic Zr_2_MoP_2_O_12_. With increasing mass ratio of Zr_2_MoP_2_O_12_, the intensity of diffraction peaks of Zr_2_MoP_2_O_12_ become much stronger. The diffraction peaks of both ZrO_2_ and Zr_2_MoP_2_O_12_ were sharp and intense, indicating their highly crystalline nature.

**Figure 2:**
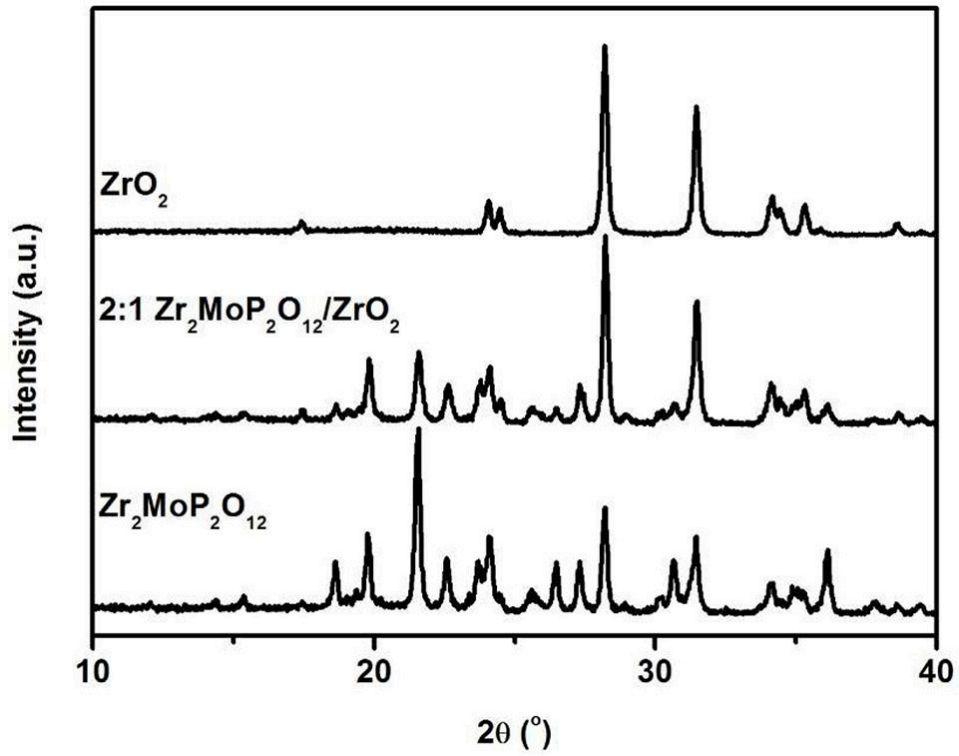
XRD patterns of the obtained 2:1 Zr_2_MoP_2_O_12_/ZrO_2_ composites sintered at 1050°C for 6 h using the Zr_2_MoP_2_O_12_ and ZrO_2_ as raw materials, and the XRD patterns of pure Zr_2_MoP_2_O_12_ and ZrO_2_ were also given for reference.

To investigate whether a chemical reaction occurred between ZrO_2_ and Zr_2_MoP_2_O_12_ during sintering at 1050°C. The 2:1 Zr_2_MoP_2_O_12_/ZrO_2_ composite was also prepared using the pure Zr_2_MoP_2_O_12_ and ZrO_2_ as raw materials. The Zr_2_MoP_2_O_12_ was mixed with ZrO_2_ at a mass ratio of 2:1 and finally calcined at 1050°C in air for 6 h. The XRD patterns of the ZrO_2_, Zr_2_MoP_2_O_12_, and 2:1 Zr_2_MoP_2_O_12_/ZrO_2_ were shown in Figure 2. Except for monoclinic ZrO_2_ and orthorhombic Zr_2_MoP_2_O_12_, no new peaks were detected, confirming that no chemical reaction occurred between ZrO_2_ and Zr_2_MoP_2_O_12_ during sintering at 1050°C.

### SEM images and density analysis

**Figure 3:**
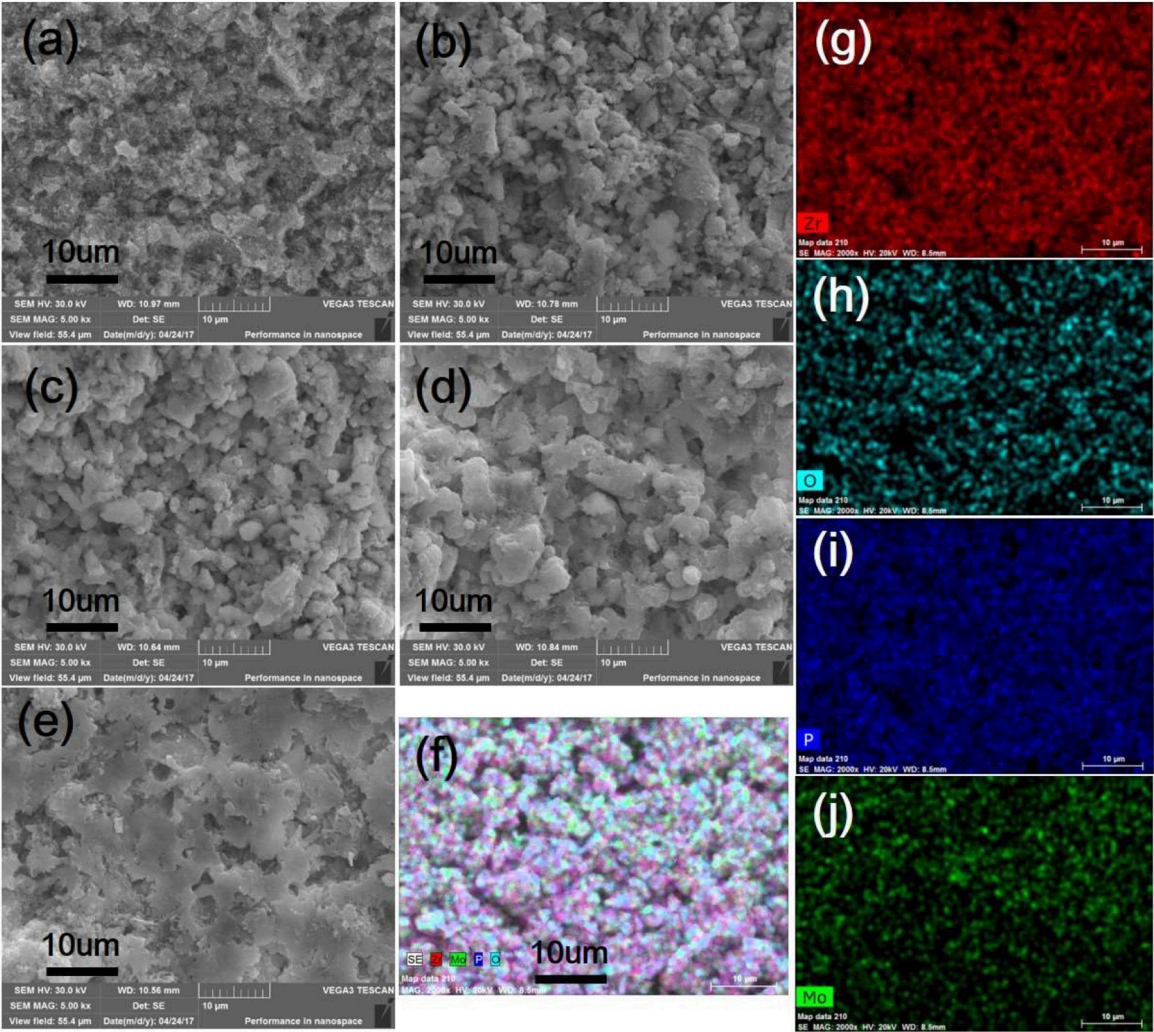
SEM images of the obtained ZrO_2_ ceramics, Zr_2_MoP_2_O_12_ ceramics and Zr_2_MoP_2_O_12_/ZrO_2_ composites with different mass ratios sintered at 1050°C for 6 h. **(a)** ZrO_2_ **(b)** Zr_2_MoP_2_O_12_:ZrO_2_ = 1:2 **(c)** Zr_2_MoP_2_O_12_:ZrO_2_ = 1:1 **(d)** Zr_2_MoP_2_O_12_:ZrO_2_ = 2:1 **(e)** Zr_2_MoP_2_O_12_ **(f)** overlaid with elemental analysis results of 2:1 Zr_2_MoP_2_O_12_/ZrO_2_ composite using EDX **(g–j)** distribution of zirconium, oxygen, phosphorus, molybdenum elements in the selected area.

Further study was carried out by the SEM analysis to identify microstructures of as-prepared Zr_2_MoP_2_O_12_, ZrO_2_, and Zr_2_MoP_2_O_12_/ZrO_2_ composites. Figure 3 shows SEM images of fracture surfaces of the pure ZrO_2_, pure Zr_2_MoP_2_O_12_ sintered bodies, and the Zr_2_MoP_2_O_12_/ZrO_2_ composites fabricated with different mass ratio of 1:2, 1:1, and 2:1. As shown in Figure 3a, as-prepared ZrO_2_ ceramics sintered at 1050°C is not compact and the ZrO_2_ grain growth is not observed obviously due to the insufficient sintering. The SEM images of the Zr_2_MoP_2_O_12_/ZrO_2_ composites fabricated at different mass ratio of 1:2, 1:1, and 2:1 are shown in Figures 3b–d. It can be seen that the Zr_2_MoP_2_O_12_/ZrO_2_ composites show nearly the same SEM images of fracture surfaces, which consist of irregular grains and some pores. With increasing content of Zr_2_MoP_2_O_12_, Zr_2_MoP_2_O_12_/ZrO_2_ ceramics became denser and displayed larger particle sizes and less porosity, suggesting that the increase of Zr_2_MoP_2_O_12_ slightly promoted the particle growth and increased the density of the Zr_2_MoP_2_O_12_/ZrO_2_ ceramics. Figure 3e shows the SEM images of fracture surfaces of the obtained pure Zr_2_MoP_2_O_12_ ceramics. The Zr_2_MoP_2_O_12_ sintered body was denser compared with the ZrO_2_ ceramics fabricated at same sintering temperature. It is compact, which is in agreement with the results reported earlier (Cetinkol et al., 2009; Isobe et al., 2016). The elemental composition and distribution of 2:1 Zr_2_MoP_2_O_12_/ZrO_2_ composite was also investigated using the EDX. The distribution of zirconium, oxygen, phosphorus, molybdenum is shown in Figures 3f–j.

### Thermal expansion properties

**Figure 4:**
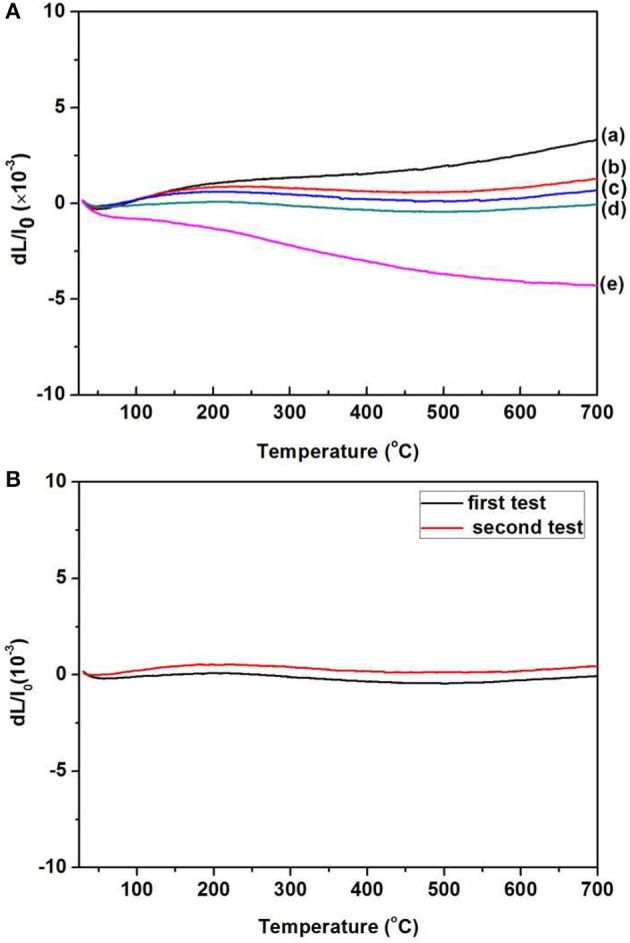
**(A)**Thermal expansion curves of the obtained ZrO_2_ ceramics, Zr_2_MoP_2_O_12_ ceramics and Zr_2_MoP_2_O_12_/ZrO_2_ composites with different mass rati006Fs sintered at 1050°C for 6 h. **(a)** ZrO_2_ **(b)** Zr_2_MoP_2_O_12_:ZrO_2_ = 1:2 **(c)** Zr_2_MoP_2_O_12_:ZrO_2_ = 1:1 **(d)** Zr_2_MoP_2_O_12_:ZrO_2_ = 2:1 **(e)** Zr_2_MoP_2_O_12_. **(B)** Cyclic thermal expansion curves of the 2:1 Zr_2_MoP_2_O_12_/ZrO_2_ composite.

**Figure 5:**
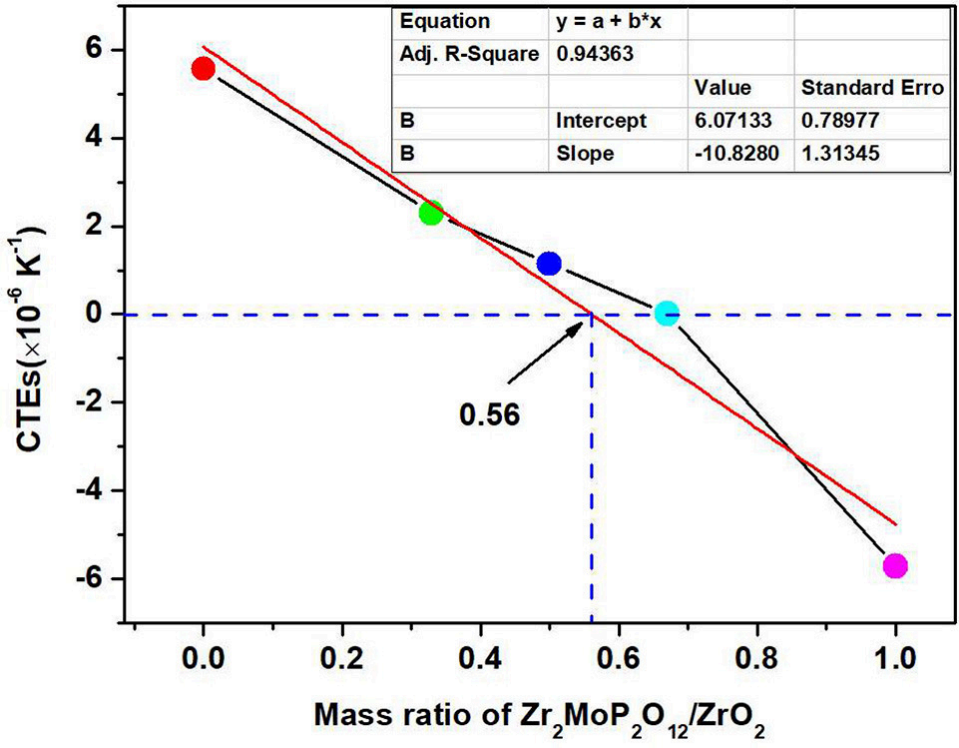
Relation between coefficients of thermal expansion and the mass ratio of the Zr_2_MoP_2_O_12_/ZrO_2_ composites sintered at 1050°C for 6 h.

For a better analysis of the thermal expansion behaviors of the composites, Figure 4A shows the thermal expansion curves of the Zr_2_MoP_2_O_12_/ZrO_2_ composites with different mass ratio calcined at 1050°C for 6 h. The thermal expansion curves of the pure Zr_2_MoP_2_O_12_ and pure ZrO_2_ ceramics are also given for reference. As shown in Figures 4A-a, Pure ZrO_2_ specimen shows positive thermal expansion in the testing temperature from 25 to 700°C with an average linear thermal expansion coefficient of 5.57 × 10^−6^ K^−1^, which agrees with literature reports (Lommens et al., 2005; Yang et al., 2007). In Figures 4A-e, it can be seen that pure Zr_2_MoP_2_O_12_ ceramics showed strong negative thermal expansion. Its average linear thermal expansion coefficient was measured to be −5.73 × 10^−6^ K^−1^ in the testing temperature range of 25–700°C. It can be found that the thermal expansion curves of the obtained samples except pure Zr_2_MoP_2_O_12_ ceramics overlap below 200°C, this abnormal behavior in the beginning of thermal expansion curves may be caused by the instrument. Above 200°C, all the samples show stable and almost linear change in the curves of thermal expansion with the increased temperature. Based on the above SEM analysis, the compact microstructure of the composites will promotes the stability of thermal expansion performance. Average linear thermal expansion coefficients of the obtained pure ZrO_2_, pure Zr_2_MoP_2_O_12_, and Zr_2_MoP_2_O_12_/ZrO_2_ composites with different mass ratios are summarized in Table 2. With increasing content of Zr_2_MoP_2_O_12_, the thermal expansion coefficient of Zr_2_MoP_2_O_12_/ZrO_2_ composite decreased gradually. The 1:2 Zr_2_MoP_2_O_12_/ZrO_2_ composite showed positive thermal expansion with a thermal expansion coefficient of 2.30 × 10^−6^ K^−1^. When the mass ratio of Zr_2_MoP_2_O_12_/ZrO_2_ decreased to 1:1, the composite also exhibited positive thermal expansion, but the CTE value decreased to 1.14 × 10^−6^ K^−1^. The 2:1 Zr_2_MoP_2_O_12_/ZrO_2_ composite showed very low thermal expansion with an average linear thermal expansion coefficient of 0.0082 × 10^−6^ K^−1^ in the temperature range of 25–700°C. Figure 4B shows the cyclic thermal expansion curves of the Zr_2_MoP_2_O_12_/ZrO_2_ composites. The two thermal expansion curves are almost the same, and the second average linear thermal expansion coefficient was test to be 0.0049 × 10^−6^ K^−1^ in the same temperature range, indicating the 2:1 Zr_2_MoP_2_O_12_/ZrO_2_ composite shows a stable thermal expansion property and the mass ratio of 2:1 (Zr_2_MoP_2_O_12_/ZrO_2_) is appropriate one. With the increase of the temperature, the volume shrinkage of the NTE Zr_2_MoP_2_O_12_ just can accommodate the volume expansion of ZrO_2_, which will keep a little change of the volume value of the 2:1 Zr_2_MoP_2_O_12_/ZrO_2_ composite. This near-zero expansion ceramic composite can withstand thermal stresses arising during sintering and subsequent quenching, which is an important criterion for a number of potential application in many fields. The results suggest that the thermal expansion coefficients of Zr_2_MoP_2_O_12_/ZrO_2_ composites can be tailored from 5.57 × 10^−6^ K^−1^ to −5.73 × 10^−6^ K^−1^ by adjusting the weight fraction of Zr_2_MoP_2_O_12_. Figure 5 shows the relation between coefficients of thermal expansion and the mass ratio of the Zr_2_MoP_2_O_12_/ZrO_2_ composites sintered at 1050°C for 6 h. The points is the data measured in the work. There is no linear relationship between the coefficients of thermal expansion and the mass ratio of the Zr_2_MoP_2_O_12_/ZrO_2_ composites. The red line is the best-fit line. According to the fitting equation, when the mass ration of Zr_2_MoP_2_O_12_/ZrO_2_ is 0.57, the Zr_2_MoP_2_O_12_/ZrO_2_ composite show zero thermal expansion. This fitting result deviates from the data obtained in the experiment. When the mass ration of Zr_2_MoP_2_O_12_/ZrO_2_ is 0.67, the near-zero thermal expansion Zr_2_MoP_2_O_12_/ZrO_2_ composite was obtained. This deviation mainly caused by the defects in the composites, such as pores and microcracks.

**Table 2:** Average linear thermal expansion coefficients of ZrO_2_ ceramics, Zr_2_MoP_2_O_12_ ceramics, and Zr_2_MoP_2_O_12_/ZrO_2_ composites with different mass ratios in corresponding testing temperature range from 25 to 700°C.

**Samples (mass ratio)**	**Coefficient of thermal expansion**
ZrO_2_	5.57 × 10^−6^ K^−1^
Zr_2_MoP_2_O_12_/ZrO_2_ = 1:2	2.30 × 10^−6^ K^−1^
Zr_2_MoP_2_O_12_/ZrO_2_ = 1:1	1.14 × 10^−6^ K^−1^
Zr_2_MoP_2_O_12_/ZrO_2_ = 2:1	0.0065 × 10^−6^ K^−1^(mean value)
Zr_2_MoP_2_O_12_	−5.73 × 10^−6^ K^−1^

## Conclusions

With the goal of thermal expansion control, NTE Zr_2_MoP_2_O_12_ was combined with the positive thermal expansion ZrO_2_ to fabricate the composites with a tailorable thermal expansion. The obtained Zr_2_MoP_2_O_12_/ZrO_2_ composites synthesized at 1050°C for 6 h were composed of orthorhombic Zr_2_MoP_2_O_12_ and monoclinic ZrO_2_, no intermediate phase was observed. With increasing content of Zr_2_MoP_2_O_12_, the thermal expansion coefficient of Zr_2_MoP_2_O_12_/ZrO_2_ composite decreased while the density of the composite increased gradually. The thermal expansion coefficients of the Zr_2_MoP_2_O_12_/ZrO_2_ composites can be tailored from 5.57 × 10^−6^ K^−1^ to −5.73 × 10^−6^ K^−1^ by changing the weight fraction of Zr_2_MoP_2_O_12_. In addition, the 2:1 Zr_2_MoP_2_O_12_/ZrO_2_ composite displayed near-zero thermal expansion with an average linear thermal expansion coefficient of 0.0065 × 10^−6^ K^−1^ in the testing temperature range of 25–700°C. This near-zero thermal expansion material will have a number of potential application in many fields due to its dimensional stability and high resistance to thermal shock.

## Author contributions

HL and ZZ designed experiments. WS, XX, and LY carried out experiments. HL, ZZ, XZ, MZ, and XC analyzed experimental results. HL and ZZ wrote the manuscript.

### Conflict of interest statement

The authors declare that the research was conducted in the absence of any commercial or financial relationships that could be construed as a potential conflict of interest.
